# Sprain of the Subaxial Cervical Spine in Childhood

**DOI:** 10.1155/2018/3653657

**Published:** 2018-11-25

**Authors:** Rebecca F. Lyons, Kevin McSorely, Mutaz Jadaan, J. P. McCabe, Colin G. Murphy

**Affiliations:** Department of Trauma and Orthopaedic Surgery, Galway University Hospitals, Galway, Ireland

## Abstract

Posterior atlantoaxial ligament disruption in children is a rare diagnosis. We present a case of a young girl with cervical spine posterior atlantoaxial ligament disruption post a fall from a climbing frame. Presenting with minimal symptoms other than neck pain, this case highlights the diagnostic difficulty and need for further radiological imaging in paediatric patients with neck pain post trauma.

## 1. Introduction

Paediatric cervical spine injuries are rare, particularly ligamentous injuries. The diagnosis of cervical spine pathology, in particular ligamentous pathology, can be difficult. This is true in the paediatric setting especially. Our case description of a young girl with a cervical spine posterior atlantoaxial ligament disruption highlights the difficulty in diagnosis as well as highlighting the sparsity of the literature on the best treatment options.

## 2. Case Description

An eight-year-old girl presented to the emergency department (ED) with cervical spine (C-spine) pain post a fall from a climbing frame approximately 4 ft off the ground. The patient had a fall from 5 ft backwards with a hyperextension injury to the neck. The patient stood from the ground holding her neck. The patient denied any weakness or paraesthesia of the upper limbs or lower limbs. No evidence of a head injury was noted, including no loss of consciousness, vomiting, or visual disturbance. The patient was immobilized by the local family practitioner and transferred to the emergency department.

An isolated cervical spine injury was identified after initial assessment. On examination, the young girl had midline C2-C5 cervical spine tenderness with associated paraspinal muscle tenderness. Neurological examination was normal.

**Figure 1:**
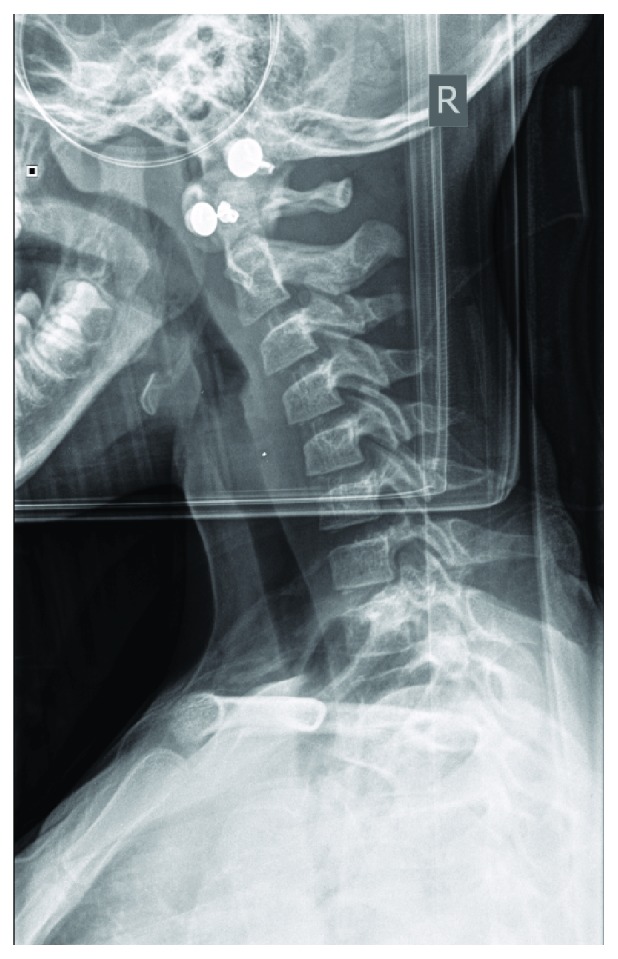
Lateral C-spine radiograph.

**Figure 2:**
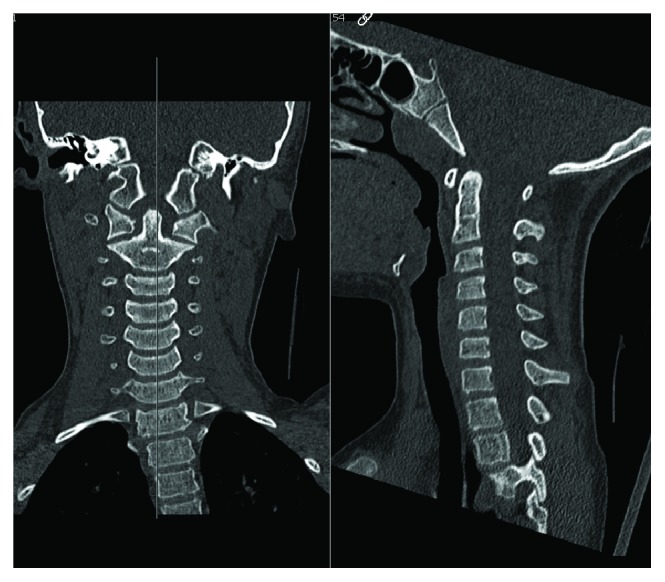
CT scan of C-spine.

Initial imaging included primary C-spine radiographs showing no bony injury (Figure 1). A computed tomography scan was also obtained, confirming no bony injury (Figure 2). The patient was admitted overnight in cervical spine immobilization for magnetic resonance imaging (MRI) due to the persistent midline tenderness.

**Figure 3:**
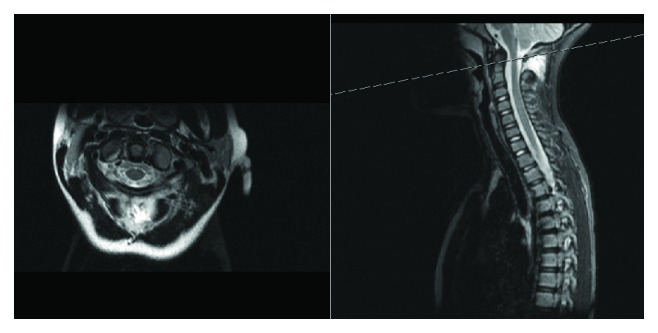
MRI scan of C-spine T1.

**Figure 4:**
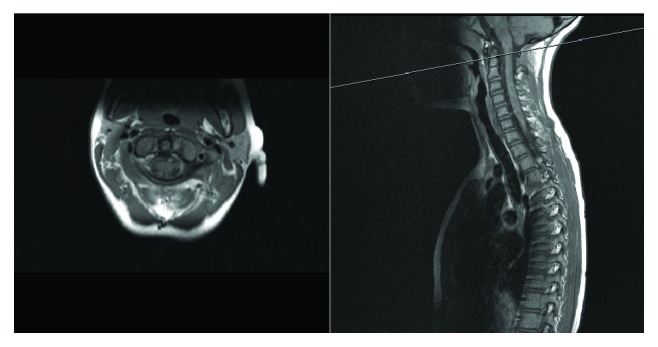
MRI of C-spine T2 stir imaging.

An MRI obtained revealed disruption of the posterior atlantoaxial ligament. No other injuries were noted including no injury to the posterior longitudinal ligament or posterior annulus fibrosus of C1-2 (Figures 3 and 4). The patient was treated in soft collar immobilization and followed up in the outpatient clinic. Follow-up cervical radiographs including flexion and extension views revealed no abnormality. Week 6 post injury, no midline tenderness was elicited on examination. Repeat radiographic imaging was normal, but static and dynamic views of the C-spine were normal with no evidence of instability. The soft C-spine immobilization was removed, and physical therapy was initiated.

## 3. Discussion

Paediatric cervical spine injuries are rare, particularly ligamentous injuries. The diagnosis of cervical spine pathology, in particular ligamentous pathology, can be difficult. This is true in the paediatric setting especially. Due to the anatomical variants combined with different injuries and biomechanical forces that are unique to the paediatric population, caution needs to be taken when assessing and reviewing radiological imaging of the patients. Up to 72% of spinal injuries in children occur in the C-spine. The upper cervical spine due to its anatomy predisposes the occiput to C2/3 level as the major site of injury [1]. This is due to the fulcrum of motion in the paediatric cervical spine at the C2-C3 level, compared to C5-C6 level in adults. Ligamentous laxity with shallow and angled facet joints as well as underdeveloped spinous processes can all contribute to higher forces acting on the C1-C2 spine. The complex ligamentous anatomy in the C1-C3 region plays a part in the stability of the spine and as highlighted in our case can be injured. Therefore, careful consideration and appropriate imaging are needed to ensure that subtle abnormalities are identified and treated [2]. Injury to the posterior atlantoaxial ligament is a rare finding in children as an isolated injury. The posterior atlantoaxial ligament is a broad, thin membrane attached to the lower border of the posterior arch of the atlas and to the upper edges of the lamina of the axis. The current literature highlights injuries involving the transverse atlantal ligament [3], and tectorial membrane injuries have been reported [4]; however, injuries to the posterior atlantoaxial ligament are not widely published. Sprains to the subaxial cervical spine in adolescents have been reported from our institution previously. McLoughlin et al. [5] demonstrated that it is possible to have a severe sprain of the C-spine in an adolescent, causing significant C-spine instability with an intact PLL and annulus at the injured level. Therefore, PLL integrity in an adolescent spine does not indicate stability. With this knowledge of the complexity of ligamentous injuries in the paediatric setting, the management of this case was with soft collar immobilization for six weeks, with serial review using dynamic radiography. From the literature, it is reported that the tectorial membrane is a critical stabilizing ligamentous structure in the Oc-C2 complex and, if it is intact, collar immobilization with close follow-up is a sufficient treatment [6, 7].

## 4. Conclusion

Posterior atlantoaxial ligament disruption in the paediatric population is rarely reported. Due to the sparsity of the literature, available diagnosis and management can be difficult for clinicians. It is imperative that all young patients presenting with persistent C-spine tenderness after a trauma are fully investigated and MR imaging undertaken. Rare ligamentous injuries must be considered in this cohort of patients due to the different biomechanics of the C-spine compared to adults. When treating these rare cases, we must use our knowledge of the spine stability. Reviewing literature on other ligamentous injuries can help guide the treatment plan for these young patients.
